# The Synthesis, Structure, and Luminescent Properties of TmMgB_5_O_10_ Crystals

**DOI:** 10.3390/ma16186084

**Published:** 2023-09-06

**Authors:** Elena A. Volkova, Victor V. Maltsev, Alexander M. Antipin, Dina V. Deyneko, Ivan V. Nikiforov, Dmitry A. Spassky, Ekaterina I. Marchenko, Diana D. Mitina, Vladimir L. Kosorukov, Vasiliy O. Yapaskurt, Daniil A. Naprasnikov, Elizaveta V. Koporulina

**Affiliations:** 1 Faculty of Geology, Moscow State University, 119991 Moscow, Russia; maltsev@geol.msu.ru (V.V.M.); marchenko-ekaterina@bk.ru (E.I.M.); varya-mitya@mail.ru (D.D.M.); kosorukov-vladimir@rambler.ru (V.L.K.); vyapaskurt@mail.ru (V.O.Y.); daniilnaprasnikov@mail.ru (D.A.N.); e_koporulina@mail.ru (E.V.K.); 2 Shubnikov Institute of Crystallography of Federal Scientific Research Centre “Crystallography and Photonics”, The Russian Academy of Sciences, 119333 Moscow, Russia; antipin@physics.msu.ru; 3 Faculty of Chemistry, Moscow State University, 119991 Moscow, Russia; deynekomsu@gmail.com (D.V.D.); nikiforoviv@my.msu.ru (I.V.N.); 4 Skobeltsyn Institute of Nuclear Physics, Lomonosov Moscow State University, 119991 Moscow, Russia; spas@srd.sinp.msu.ru; 5 Institute of Physics, University of Tartu, 50411 Tartu, Estonia; 6 Melnikov Research Institute of Comprehensive Exploitation of Mineral Resources, The Russian Academy of Sciences, 111020 Moscow, Russia

**Keywords:** borates, flux growth, crystal structure, electronic band, differential scanning calorimetry, photoluminescence

## Abstract

TmMgB_5_O_10_ spontaneous crystals were synthesized via the flux-growth technique from a K_2_Mo_3_O_10_-based solvent. The crystal structure of the compound was solved and refined within the space group *P*2_1_/*n*. The first principles calculations of the electronic structure reveal that TmMg-pentaborate with an ideal not defected crystal structure is an insulator with an indirect energy band gap of approximately 6.37 eV. Differential scanning calorimetry measurements and powder X-ray diffraction studies of heat-treated solids show that TmMgB_5_O_10_ is an incongruent melting compound. A characteristic band of the Tm^3+^ cation corresponding to the ^3^H_6_ → ^1^D_2_ transition is observed in the photoluminescence excitation spectra of TmMg-borate. The as-obtained crystals exhibit intense blue emission with the emission peaks centered at 455, 479, 667, and 753 nm. The most intensive band corresponds to the ^1^D_2_ → ^3^F_4_ transition. TmMgB_5_O_10_ solids demonstrate the thermal stability of photoluminescence.

## 1. Introduction

In recent decades, a large amount of research has focused on the development of materials used as environmentally friendly light sources and phosphors, as well as laser and nonlinear optical materials. Future progress in science and technology is directly related to the search and further application of new compounds, as well as to the improvement of the parameters of existing materials. One of the promising classes of such compounds is rare-earth borates, which demonstrate high chemical stability, thermal and radiation resistance, wide transparency area, high laser threshold, etc. In addition, borates have a wide variety of chemical compositions and crystal structures due to the ability of the boron atom to form various anionic and polyanionic groups [1]. Nowadays, the search for borate materials that can be used as phosphors is an actual problem. There are many borate crystals that do not exhibit luminescent properties in their pure form, and their luminescent characteristics are associated with doping, such as the incorporation of rare-earth or transition metal cations into their structure.

In particular, among numerous borates, a group of rare-earth magnesium pentaborates can be used as phosphors to create powerful emitters of the visible range with ultraviolet (UV) excitation [2,3]. Rare-earth magnesium borates with the general chemical formula *Ln*MgB_5_O_10_ (where *Ln* = La, Ce-Nd, and Sm-Er) were first synthesized by Saubat et al. in 1980 by the solid-phase method. LaMgB_5_O_10_ single crystals were then obtained by melting a mixture of La_2_O_3_, MgO, and B_2_O_3_ at 1200 °C with an excess of magnesium and boron oxides with respect to the stoichiometric amount to compensate for volatilization losses. The crystal structure of LaMgB_5_O_10_ was solved and refined within the space group (sp. gr.) *P*2_1_/*c* [4]. The powder patterns of other polycrystalline *Ln*MgB_5_O_10_ samples were also indexed in the same space group. These materials exhibit remarkable structural features: a weak atomic *Ln*/O ratio, well-isolated *Ln* chains separated by large polyborate anions, and a highly covalent matrix suggest the possibility of relatively low-concentration quenching. *Ln*MgB_5_O_10_ is expected to be an excellent material as a phosphor host and a matrix for solid-state lasers.

The luminescent properties of this group of compounds were first described by the authors of [5,6] for LaMgB_5_O_10_ alloyed with Eu^3+^, Tb^3+^, and Ce^3+^. The luminescence parameters of Ce^3+^:LaMgB_5_O_10_ and Ce^3+^:YMgB_5_O_10_ powders [7], nanocrystalline (La,Gd)MgB_5_O_10_:Ce^3+^/Tb^3+^ thin films synthesized by the sol–gel method [8], and LaMgB_5_O_10_-based glasses, doped with Ce^3+^, Tb^3+^, and Mn^2+^ were studied later [9]. Recently, data on the luminescence of LaMg-pentaborate crystals doped with Tb^3+^ and Eu^3+^ ions were obtained by the authors of Ref. [3]. There are also a number of investigations on the characterization of large-size and high-quality LaMgB_5_O_10_, GdMgB_5_O_10_, and YMgB_5_O_10_ laser crystals doped with Yb^3+^, Er^3+^, and Nd^3+^ (see, for example, Refs. [5,6,7,8,9]). Based on an analysis of spectra and laser characteristics, it was shown that these borates are promising candidates for multi-wavelength laser crystals. 

A review of previous studies on the synthesis and crystal growth of rare-earth pentaborates shows that the Li_2_O–B_2_O_3_–LiF mixed flux is the most commonly used to obtain *Ln*MgB_5_O_10_ compounds by the solution growth on dipped seeds technique [10,11]. Recently, GdMgB_5_O_10_ single crystals were obtained from K_2_Mo_3_O_10_ flux [12]. The authors of Ref. [13] performed experiments in Li_2_O–B_2_O_3_–LiF- and K_2_Mo_3_O_10_-based systems to determine the most suitable one for the growth of *Ln*MgB_5_O_10_ bulk crystals. It was shown that the K_2_Mo_3_O_10_ flux is preferable. The strong tendency of Li_2_O-B_2_O_3_-LiF melts toward glass formation, and the high volatility and reactivity of fluorides at high temperatures make reproducible growth of high-quality crystals difficult. It was also proposed to use pre-synthesized *Ln*MgB_5_O_10_ tablets as a crystal-forming agent instead of the mixture of corresponding *Ln*_2_O_3_–MgO–B_2_O_3_ oxides. 

Spontaneous TmMgB_5_O_10_ (TmMB) single crystals were obtained for the first time in the framework of the approach described in [13]. The present work is focused on the synthesis and study of the crystal structure, thermal behavior, and luminescent properties of TmMgB_5_O_10_ crystals.

## 2. Materials and Methods

Two subsequent routes were applied to obtain TmMg-borate: solid-state synthesis and flux-growth techniques. In both cases, a vertical resistance-heated furnace equipped with a Proterm-100 precision temperature controller and a set of Pt/Rh-Pt thermocouples was used. Tm_2_O_3_ (99.996%), H_3_BO_3_ (A.C.S. grade), and MgO (A.C.S. grade) were used as crystal-forming agents for the solid-state synthesis of polycrystalline TmMB. Calculated amounts of Tm_2_O_3_, MgO, and H_3_BO_3_ were weighted, mixed together, and pressed into tablets 15 mm in diameter. The compact TmMB tablets were heated in an alundum crucible at the temperature of 900 °C for 72 h, and then a furnace was gradually cooled to room temperature.

In solid-phase synthesis, crystal-forming components were initially weighed corresponding to the composition of TmMgB_5_O_10_. As a result, only the TmBO_3_ phase was formed. According to the Le Chatelier principle, an excess of one of the components shifts the direction of the reaction toward the formation of the desired phase. Further experiments were carried out using a non-stoichiometric load with 100% excess of MgO. This approach led to the formation of a nearly monophase TmMgB_5_O_10_ sample at 900 °C.

Spontaneous TmMB crystals were obtained from a high-temperature K_2_Mo_3_O_10_-based flux melt. The K_2_Mo_3_O_10_ solvent used was a stoichiometric mixture of K_2_MoO_4_ (A.C.S. grade) and MoO_3_ (A.C.S. grade). TmMB previously synthesized by the solid-state technique was taken as a crystal-forming component. A thoroughly mixed starting charge with a crystal phase-to-flux weight ratio of 30:70 wt.% was loaded into a 15 mL platinum crucible, placed in a furnace, heated to 900 °C and held for 24 h to ensure complete homogenization of the solution. Subsequently, the flux melt was cooled to 800 °C at a rate of 1 °C/h, followed by cooling at 10 °C/h to 300 °C.

K_2_Mo_3_O_10_-based solvents seem to be the most suitable for the growth of different borate crystals. Nevertheless, *Ln*MgB_5_O_10_ solids dissolve incongruently in a K_2_Mo_3_O_10_ melt, and reactions between K_2_Mo_3_O_10_ and *Ln*MgB_5_O_10_ result in new crystalline phases, mostly crystal-forming oxides, as well as *Ln*BO_3_. The formation temperatures of these co-crystallizing phases depend on the borate type and its concentration in a flux melt. Small crystals of co-existing phases that appeared at high temperatures become the nucleation centers of spontaneous *Ln*MgB_5_O_10_ crystals upon cooling. As a result of the incongruent dissolution of *Ln*Mg-borate, the melt is enriched in B_2_O_3_ and *Ln*_2_O_3_. Rare-earth metal oxides, in turn, cause the formation of *Ln*BO_3_ with calcite- or vaterite-type structures depending on the temperature [14]. The temperature range for *Ln*MB synthesis is restricted by the properties of K_2_Mo_3_O_10_-based high-temperature solutions and by borate stability in such a melt. Usually, the melting temperature of the charge does not exceed 1050 °C because of the significant increase in the borate decomposition rate. The lower temperature boundary (commonly ~800 °C) is determined by a significant increase in melt viscosity with a corresponding decrease in the crystal growth rate.

The morphological feature and elemental analysis were performed by analytical scanning electron microscopy (SEM) technique using a JSM-IT500 microscope, JEOL Ltd., Tokyo, Japan, equipped with energy dispersive X-ray (EDX) detector X-Max-50, Oxford Instruments Ltd. (Abingdon, UK), GB (Laboratory of Analytical Techniques of High Spatial Resolution, Dept. of Petrology, Moscow State University).

Single-crystal X-ray studies were carried out using an Xcalibur diffractometer equipped with a CCD-detector (MoKα radiation). The XRD data were integrated using the CrysAlisPro 1.171.41.119a program software [15]. Powder X-ray diffraction (PXRD) studies were carried out on a Rigaku MiniFlex-600 powder diffractometer (Rigaku Corp., Tokyo, Japan). PXRD datasets were collected in continuous mode at room temperature (CuKα radiation) in the range of 2*θ* = 3–90°, and a scan speed of 4° per minute. 

Differential scanning calorimetry (DSC) measurements were performed by means of a STA 449 F5 Jupiter (Netzsch, Selb, Germany) in the temperature range of 50–1200 °C with a heating rate of 20 °C/min in Ar gas flow. A PtRh20 crucible of 85 μL volume was used in the DSC experiments.

The band structure for TmMB was calculated within the framework of density functional theory using the pseudopotential plane wave basis of the Quantum Espresso v. 7.1 software package [16]. The electronic exchange correlations were treated by the Perdew–Burke–Ernzerhof (PBE) approach under a generalized gradient approximation (GGA) [17]. Ultrasoft pseudopotentials were used to describe the interaction between electrons and ions [18]. The kinetic energy cut-off values of the wavefunctions were limited to 58 Ry. The integration calculation of the system in the Brillouin region uses the Monkhorst–Pack scheme, the k grid point is 3 × 3 × 3, and the cut-off energy of the plane wave of the system is set to 750 eV to ensure the convergence of energy and the configuration of the system at the level of quasi-complete plane wave base. In the self-consistent field operation, the Pulay density mixing method is adopted, and the self-consistent field is set to 1 × 10^−6^ eV/atom. The calculations did not include spin–orbit coupling. Full relativistic effects were taken for the nucleus states, and the scalar relativistic approximation was used for the valence states.

A Cary Eclipse (Agilent Technologies) fluorescence spectrometer equipped with a 75 kW xenon light source (pulse length τ = 2 μs, pulse frequency *ν =* 80 Hz, wavelength resolution 0.5 nm; PMT Hamamatsu R928) was used to record photoluminescence emission (PL) and excitation (PLE) spectra. All measurements were performed at room temperature and corrected for the sensitivity of the spectrometer. The quantum yield defined as the ratio of the number of emitted photons to the number of photons absorbed (QY, %) for the visible region was measured on an Edinburgh Instruments FS5 spectrofluorometer equipped with a SC-30 integrating sphere module and a Hamamatsu PMT R928P. The measurement was performed at room temperature. 

Luminescence emission spectra under heating upon 500 K with the excitation in the UV region were measured using a 150 W xenon lamp (Oriel Instruments, Stratford, ON, USA) as an excitation source, an MDR-206 primary monochromator (Lomo, Saint Petersburg, Russia), and a LOT-Oriel MS-257 spectrograph (Oriel Instruments, Stratford, ON, USA) equipped with a Marconi CCD detector (Marconi Applied Technologies Limited, Chelmsford, UK). Samples were mounted into a Cryotrade LN-120 vacuum optical cryostat (Cryotrade Engineering, Moscow, Russia).

## 3. Results and Discussion

**Figure 1:**
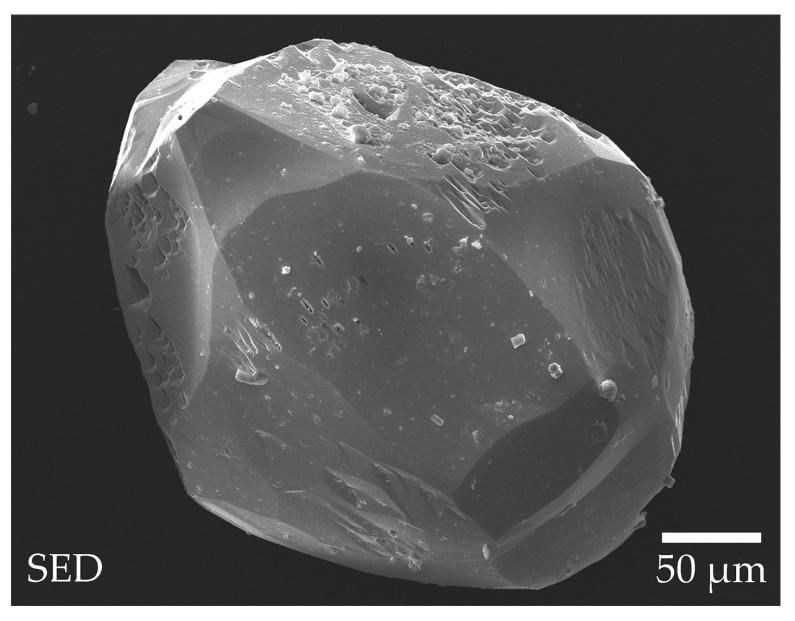
TmMgB_5_O_10_ spontaneous crystals grown from a K_2_Mo_3_O_10_-based system.

Spontaneous isometric crystals up to 1 mm in size were obtained from a K_2_Mo_3_O_10_-based flux melt (Figure 1). Preliminary XRD studies were performed to find the sample with the best diffraction reflection profile for a single-crystal X-ray study. Based on these pre-experiments, all tested specimens were crystal clusters or twin crystals.

**Figure 2:**
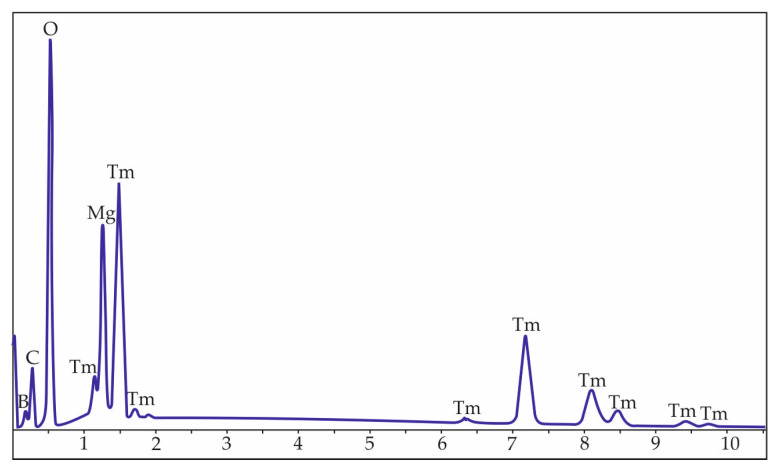
Energy-dispersive X-ray qualitative analysis of TmMg-borate crystal.

Qualitative energy-dispersive X-ray analysis showed peaks corresponding to thulium, magnesium, boron, and oxygen (Figure 2).

**Figure 3:**
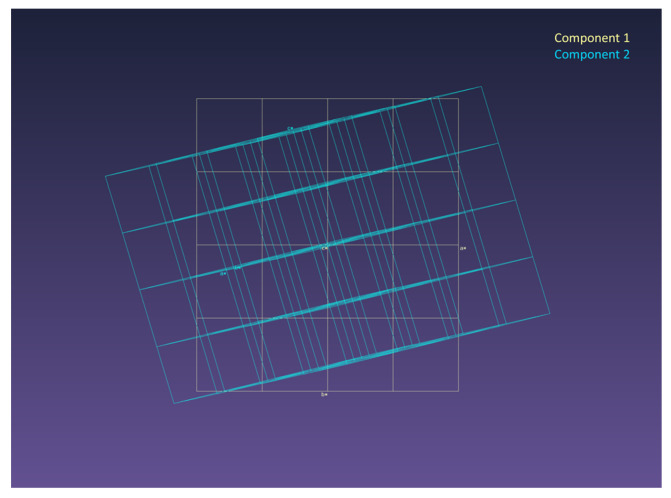
Mutual orientation of two monoclinic components of Tm in reciprocal space.

The diffraction patterns of TmMgB_5_O_10_ were analyzed using the CrysAlisPro software v. 1.171.41.119a [15]. As a result, reflexes corresponding to two components with close unit cell parameters *a*_1_ = 8.476(1) Å, *b*_1_ = 7.577(1) Å, *c*_1_ = 9.368(1) Å, *a*_2_ = 8.474(2) Å, *b*_2_ = 7.572(2) Å, and *c*_2_ = 9.361(2) Å were revealed. No rigid regularities of the mutual orientation of the bases of these components, associated with a significant change in the size of the unit cell or sp. gr. (superstructure), were found (Figure 3).

The relationship between the components can be represented by the following vector equations:

*a* = (−0.711 × *a*′ − 0.29 × *b*′ + 0.547 × *c*′);

*b* = (0.578 × *a*′ + 0.136 × *b*′ + 0.644 × *c′*);

*c* = (−0.258 × *a*′ − 1.199 × *b*′ − 0.01 × *c*′).

The ratio of the two twin components is 75.1% and 23.0% of all registered reflections, respectively. The existence of two components for the TmMB single crystal slightly reduces the quality of the obtained structural model and leads to overestimated structure refinement factors. The model proposed is obtained from the experimental data of the first component (75.1% of the total number of reflections). It is not possible to correctly solve and refine the crystal structure of the second component due to the extremely high value of the parameter R_int2_ = 79%.

The crystal structure model was determined and refined using the Jana2020 software package [19]. Structural patterns similar to those described in the literature [20,21] were obtained using the SuperFlip utility [22] within the *sp*. *gr*. *P*2_1_/*n*. The experimental and crystallographic data and structural refinements of TmMB are summarized in Table 1. 

The model of the structure consists of 17 independent atomic positions: one thulium position (Tm1), one magnesium position (Mg1), five boron atom positions (B1–B5), and ten oxygen positions (O1–O10). The low quality of the crystal and, as a result, of the diffraction experiment does not allow one to refine the parameters of anisotropic atomic displacements, which affects the value of the structure refinement factors and the values of the residual electron density. The atomic displacement parameters were refined isotropically for all localized positions; the occupancy of all positions is 100% (Table 2). The principle interatomic distances are listed in Table 3.

**Figure 4:**
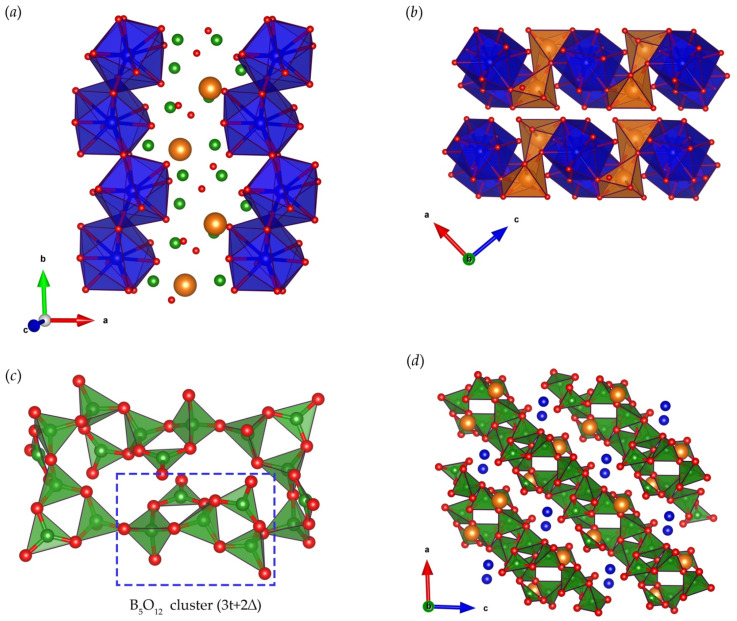
Structure of TmMgB_5_O_10_: (**a**) zigzag chains of edge-sharing TmO_10_ distorted polyhedral extending along the *b* axis; (**b**) Tm-Mg-O layers, formed by linked Tm-O chains and dimeric Mg_2_O_10_ clusters, projection along the *b* axis; (**c**) 4-membered ring assembled from B_5_O_12_ neighboring clusters; (**d**) two-dimensional B-O layers, linked together by Tm and Mg cations, projection along *b* axis. The blue, orange, green, and red balls represent Tm, Mg, B, and O, respectively. Visualization by VESTA [23].

The thulium cations are coordinated by ten O atoms, forming distorted polyhedra that share a common edge and form one-dimensional zigzag chains extending along the *b* axis (Figure 4a). Magnesium atoms are located inside oxygen octahedra that form edge-sharing dimeric Mg_2_O_10_ groups. The adjacent Tm-O chains are linked together by dimeric Mg_2_O_10_, building Tm-Mg-O layers (Figure 4b). Boron atoms have a mixed coordination (3t + 2∆): Three B atoms are located inside oxygen tetrahedra and the other two B atoms are coordinated by three O atoms, forming a planar triangle. BO_4_ tetrahedra and BO_3_ triangles connected by corners form a pentaborate cluster [1]. Each B_5_O_12_ unit via a corner-sharing O atom between two neighboring clusters forms 4-membered rings, from which infinite two-dimensional B-O layers are further assembled (Figure 4c). Countless boron–oxygen layers are linked together by Tm and Mg cations forming a rigid three-dimensional framework (Figure 4d).

**Figure 5:**
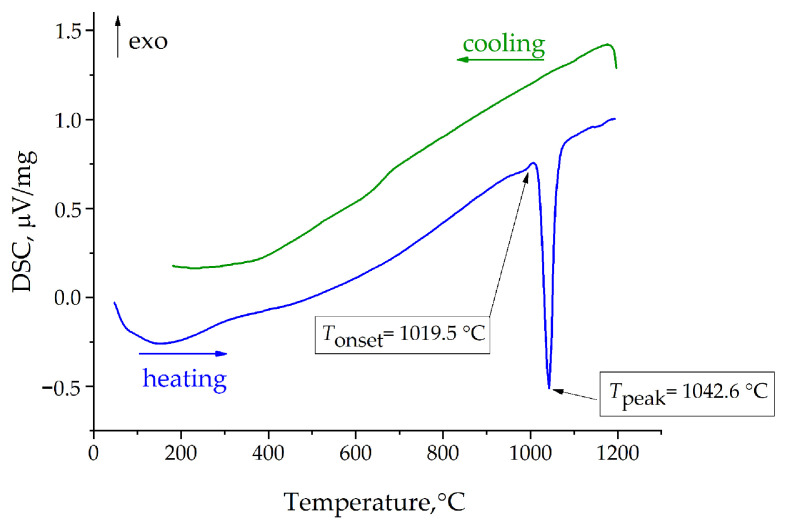
DSC curve of TmMgB_5_O_10_ compound in the temperature range of 50–1200 °C.

To determine the behavior and melting temperature of TmMg-borate, DSC measurements were performed. The calorimetric data in the temperature range of 50–1200 °C are shown in Figure 5. The DSC heating curve for the sample features one sharp endothermic peak with an onset temperature of ~1020 °C, but no exothermal peak was observed on the cooling curve. After melting, the residue was characterized by PXRD analysis, which showed that it was different from the initial compound powder, and the decomposition products of TmMB compound are a mixture of TmBO_3_ and Mg_2_B_2_O_5_. These results agreed with those previously obtained for Yb:YMgB_5_O_10_ crystals [24]. 

**Figure 6:**
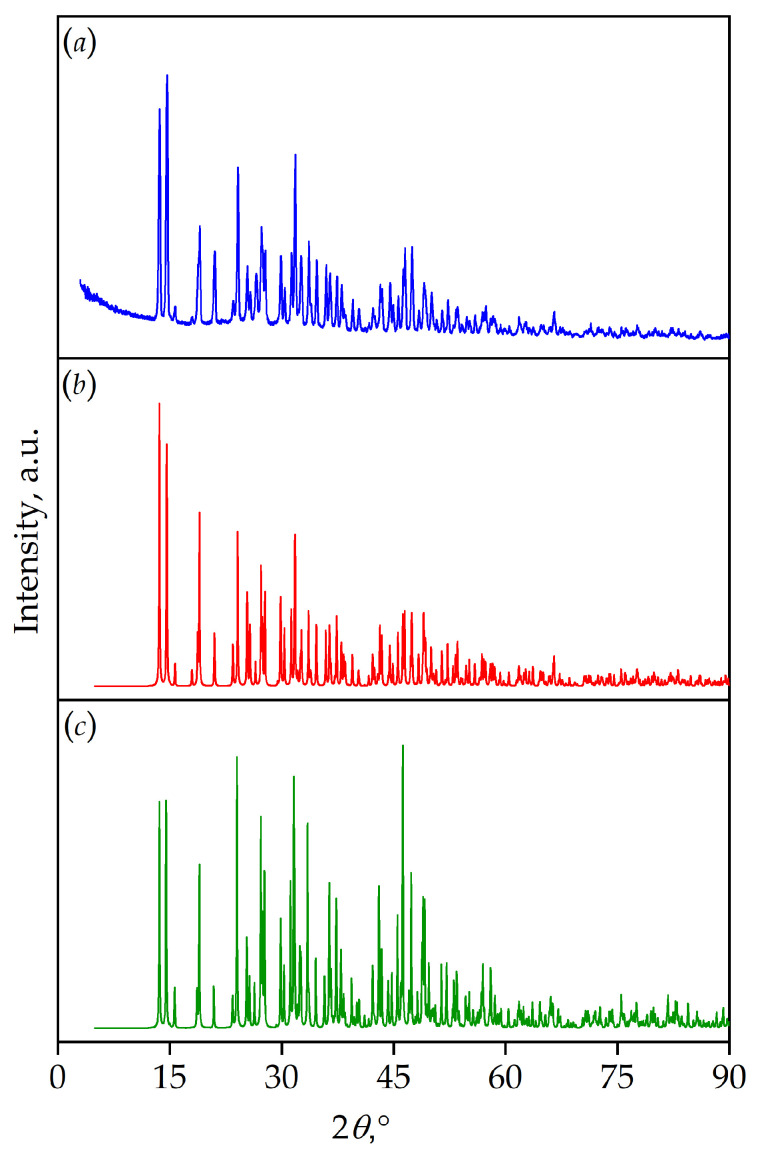
PXRD patterns (**a**) of TmMgB_5_O_10_ solids, (**b**) calculated from the cif file obtained from the structural studies of a single crystal, and (**c**) calculated from the cif file of YMgB_5_O_10_ (ICSD #4489).

The PXRD dataset was collected to confirm that the crystal structure is representative of the entire experimental sample. The pattern fits well with that calculated from the cif file obtained from the structural studies and calculated from the cif file of YMaB_5_O_10_ (ICSD #4489) (Figure 6).

**Figure 7:**
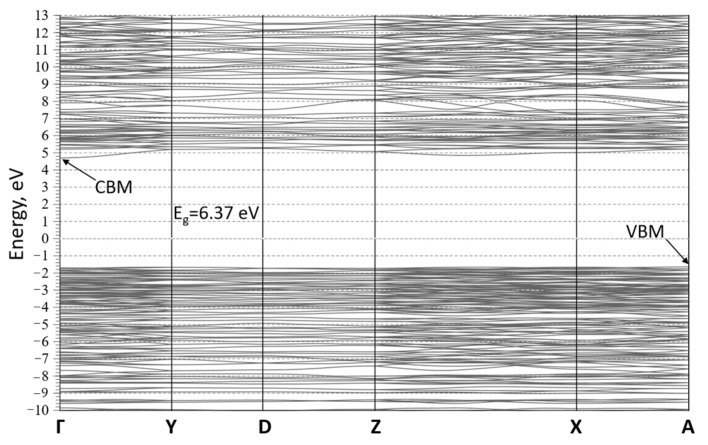
The calculated band structure of MgTmB_5_O_10_.

The band structure of the TmMB material was calculated to find out the electronic characteristics of the TmMgB_5_O_10_ model structure (Figure 7). The outcome of this analysis shows that TmMB is an insulator, as evidenced by the determination of an indirect energy gap of about 6.37 eV. This gap was determined by the A point at the maximum of the valence bands (VBM) and the Γ point at the minimum of the conduction band (CBM). However, it should be noted that GGA calculations have been shown to underestimate band gaps, and therefore, the experimental band gap for MgTmB_5_O_10_ may differ slightly from the calculated value. Notably, the VBM is a flat band, and the maximum values at Γ and A points in the Brillouin zone are quite close. This suggests that even a small concentration of defects in the simulated crystal structure could result in a direct band gap at the Γ point.

**Figure 8:**
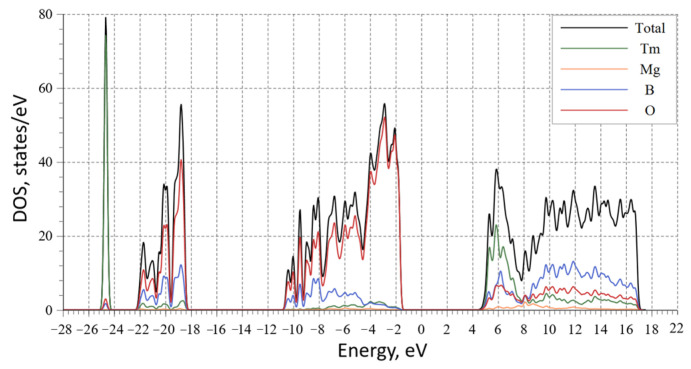
The calculated density of states of MgTmB_5_O_10_.

According to the calculated density of states (DOS) of MgTmB_5_O_10_ (Figure 8), the electronic structure in the energy range from −28.0 to 22 eV mainly comprises the Tm-*s* and O-*s/p* states with smaller contributions from the B-*s/p* and Mg-*s/d/p* states. The significant contribution to the lower conduction band comes from the Tm-*d* state, while O-*s* in the upper VB has a considerable effect on the dispersion.

**Figure 9:**
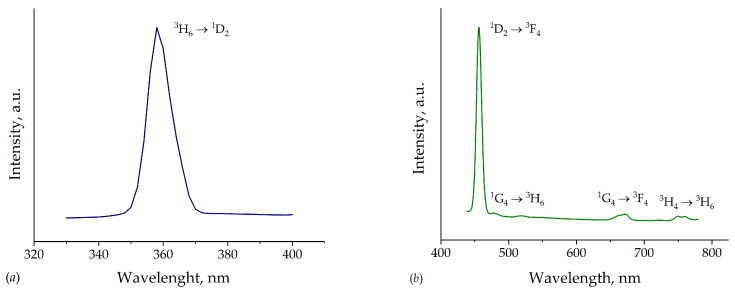
(**a**) PLE (λ_em_ = 455 nm) and (**b**) PL (λ_em_ = 358 nm) spectra for MgTmB_5_O_10_.

**Figure 10:**
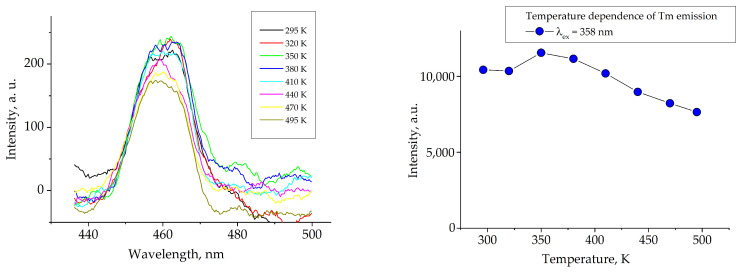
The temperature dependence of Tm^3+^ photoluminescence (λ_em_ = 358 nm); the right figure shows the relative emission intensity as a function of temperature.

The normalized photoluminescence excitation and photoluminescence emission spectra of the TmMgB_5_O_10_ compound are shown in Figure 9. On the PLE spectrum (Figure 9a) for TmMgB_5_O_10_ the typical transition of the Tm^3+^ ion is observed in the near-ultraviolet region. The sharp line with maxima centered at 358 nm corresponds to the transition ^3^H_6_ → ^1^D_2_ [25]. Since the indirect energy gap is about 6.37 eV, excitation with such higher transition energies is not possible in the 220–340 nm range. The standard sharp lines for Tm^3+^ were registered for the PL spectrum (Figure 9b). The maxima centered at 455, 479, 667, and 753 nm correspond to the _1_D_2_ → ^3^F_4_, ^1^G_4_ → ^3^H_6_, ^1^G_4_ → ^3^F_4_, and ^3^H_4_ → ^3^H_6_ transitions according to [26]. The most intensive transition is observed for ^1^D_2_ → ^3^F_4_, which emits in the blue region. So, MgTmB_5_O_10_ demonstrates photoluminescence in the blue spectral region. Additionally, the integral intensity is significantly low and takes a few arb. units. Such behavior can be explained by the presence of a high concentration quenching effect in observed TmMgB_5_O_10_, which was registered in other hosts (see, for example, Refs. [27,28]. This is confirmed by the minor value of QY of approximately 3%. Such extremely low QY can indicate concentration quenching through all the resonance channels of cross-relaxation “upward” and “downward” [29] in a studied host. The calculated Tm–Tm distance in the structure (approximate value is 3.94 Å) leads to registered concentration quenching. However, in spite of DFT calculations of the MgTmB_5_O_10_ band structure for an ideal crystal model showing that this material is an insulator, contrasting these results with the experimental observations revealed the presence of PL peaks in the blue spectral range. Hence, it can be inferred that the presence of point defects in the crystal structure gives rise to additional electronic states. A similar conclusion was observed in [20], where the properties of the MgYB_5_O_10_ compound were investigated. Furthermore, observed crystals exhibit thermal stability of photoluminescence (Figure 10). As the temperature increases, the total integral intensity of the ^1^D_2_ → ^3^F_4_ transition decreases slowly, indicating the standard thermal quenching of photoluminescence for MgTmB_5_O_10_. The thermal stability of photoluminescence in this Tm^3+^-activated host is higher than in other hosts, for example, in crystals BaY_2_F_8_ [30] and powder YNbO_4_ [31]. It should be noted that other transitions in temperature dependence were not detected because of their low intensity.

## 4. Conclusions

Spontaneous crystals of TmMgB_5_O_10_ were synthesized by a two-step technique, where polycrystalline TmMB solids, previously obtained by solid-phase synthesis, were used as a crystal-forming agent for flux growth from a K_2_Mo_3_O_10_-based system. The obtained solids crystallize in the sp. gr. *P*2_1_/*n* with unit cells parameters *a* = 8.476(1) Å, *b* = 7.577(1) Å, *c* = 9.368(1) Å, *β* = 94.035(3)°, *V* = 600.1(1) Å^3^, and z = 4. The electronic structure of TmMgB_5_O_10_ was calculated. A comprehensive study of TmMg-borate crystals was performed using scanning electron microscopy, the DSC technique, and luminescence spectroscopy.

## Figures and Tables

**Table 1:** Crystallographic data, details relating to X-ray data collection, and structure refinements of TmMgB_5_O_10_.

Chemical Formula	TmMgB_5_O_10_
*sp. gr.*, Z	*P*2_1_/*n*, 4
*a*, *b*, *c*, Å	8.476(1) 7.577(1) 9.368(1)
*β*, °	94.035(3)
*V*, Å	600.1(1)
*D*, g/cm^3^	4.508
Radiation; *λ*, Å	MoK_α_; 0.71069
*μ*, mm^−1^	14.946
*T*, K	295
Diffractometer	Xcalibur
Scan type	ω
Absorption coefficient; T_min_/T_max_	By cut; 0.125/1.000
*θ*_max_, °	30.61
Limitation of *h*, *k*, *l*	–11 ≤ *h* ≤ 12; –10 ≤ *k* ≤ 10; –12 ≤ *l* ≤ 13
The number of reflections: measured/independent (*N*1)/I > 3σ(I) (*N*2)	8445/1601/1303
*R*_int_, %	19.2
The method of refinement	OLS by F2
The number of refined parameters	71
Consideration of extinction, *k*	Type 2
*R_1_*/*R_w2_* on *N*1	0.0543/0.1045
*R_1_*/*R_w2_* on *N*2	0.0638/0.1056
*S*	2.42
Δ*ρ*_min_/Δρ_max_	–5.54/5.50

**Table 2:** Atomic coordinates and equivalent isotropic thermal parameters *U*_eq_ in the TmMgB_5_O_10_ structure.

Atom	*x*/*a*	*y*/*b*	z/c	U_eq_, Å^2^
Tm1	0.3152(1)	0.6865(1)	0.2582(1)	0.0065(1)
Mg1	0.596(1)	0.406(1)	0.130(1)	0.005(1)
B1	0.335(1)	0.898(1)	0.491(1)	0.006(1)
B2	0.436(1)	0.186(1)	−0.089(1)	0.006(1)
B3	−0.084(1)	0.573(1)	0.254(1)	0.008(1)
B4	0.517(1)	0.676(1)	0.602(1)	0.003(1)
B5	0.224(1)	−0.034(1)	−0.056(1)	0.006(1)
O1	0.250(1)	0.945(1)	0.370(1)	0.006(1)
O2	0.320(1)	0.124(1)	−0.011(1)	0.007(1)
O3	0.812(1)	0.532(1)	0.121(1)	0.006(1)
O4	0.682(1)	0.150(1)	0.140(1)	0.006(1)
O5	0.451(1)	0.770(1)	0.475(1)	0.007(1)
O6	0.508(1)	0.350(1)	−0.073(1)	0.006(1)
O7	0.490(1)	0.093(1)	−0.201(1)	0.008(1)
O8	0.583(1)	0.472(1)	0.351(1)	0.007(1)
O9	0.038(1)	0.705(1)	0.228(1)	0.002(1)
O10	0.677(1)	0.611(1)	0.580(1)	0.007(1)

**Table 3:** The main interatomic distances (Å) in the TmMgB_5_O_10_ structures.

Atoms	Distance, Å	Atoms	Distance, Å
Tm1–O1	2.304(7)	B2–O2	1.347(13)
Tm1–O1	2.236(7)	B2–O6	1.387(12)
Tm1–O2	2.747(7)	B2–O7	1.363(13)
Tm1–O5	2.354(7)	<B2–O>	1.365
Tm1–O6	2.389(7)	B3–O3	1.512(12)
Tm1–O7	2.441(7)	B3–O4	1.456(13)
Tm1–O8	2.875(6)	B3–O7	1.452(13)
Tm1–O9	2.355(6)	B3–O9	1.467(12)
Tm1–O10	2.713(7)	<B3–O>	1.472
Tm1–O10	2.497(1)	B4–O5	1.462(12)
<Tm1–O>	2.4911	B4–O8	1.492(12)
Mg1–O3	2.067(7)	B4–O9	1.485(12)
Mg1–O4	2.071(8)	B4–O10	1.472(12)
Mg1–O6	2.040(7)	<B4–O>	1.478
Mg1–O6	2.104(7)	B5–O2	1.490(12)
Mg1–O8	2.137(7)	B5–O4	1.455(13
Mg1–O9	2.371(7)	B5–O8	1.504(11)
<Mg1–O>	2.131	B5–O10	1.483(13)
B1–O1	1.347(12)	<B5–O>	1.483
B1–O3	1.353(13)		
B1–O5	1.398(12)		
<B1–O>	1.366		

## Data Availability

Cambridge Crystallographic Data Centre. CCDC reference 2286790.
